# Fungicidal properties of ginger (*Zingiber officinale*) essential oils against *Phytophthora colocasiae*

**DOI:** 10.1038/s41598-022-06321-5

**Published:** 2022-02-09

**Authors:** Muhammad Talib Kalhoro, Hong Zhang, Ghulam Mujtaba Kalhoro, Fukai Wang, Tianhong Chen, Yahya Faqir, Farhan Nabi

**Affiliations:** School of Life Science and Engineering, Southwest University of Science and Technology, Mianyang, 621000 Sichuan China

**Keywords:** Biological techniques, Plant sciences

## Abstract

Recently, plant essential oils (EOs) have attracted special attention in plant disease control and food preservation. Since ancient times, essential oils extracted from plants have exhibited many biological characteristics, especially antimicrobial properties. Recent studies have described the potentials of EOs and derivatives to inhibit the growth and reproduction of microorganisms, mainly in response of overwhelming concerns of consumers about food safety. In the context of returning to nature, with the advancement of science and technology and improved living standards, people have begun to seek solutions for food hygiene without chemical additives. Therefore, biological pesticides and plant-oriented chemicals have received special attention from scientists because they are environmentally friendly and nonhazardous, sustainable, and effective alternatives against many noxious phytopathogens. Present study is intended to appraise the fungicidal properties of ginger EOs to combat leaf blight disease of taro, which threatens global taro production. Farmers often hinge on extremely toxic synthetic fungicides to manage diseases, but the residual effects and resistance of chemicals are unavoidable. The microwave-assisted hydrodistillation method was used for ginger EOs extraction and an FTIR (ATR) spectrometer was used to evaluate their chemical composition and citral was identified as most abundant compound (89.05%) in oil. The pathogen isolated from lesions of diseased taro plants was identified as *Phytophthora colocasiae* and used as test fungus in the present study. Ginger EO was evaluated in-vitro for antifungal properties against mycelium growth, sporangium production, zoospore germination, leaf, and corm necrosis inhibition. Repeated experiments have shown that the concentration of ginger essential oil (1250 ppm) proved to be the lowest dose to obtain 100% inhibition of fungal growth and spore germination, sporangia formation and leaf necrosis assessment. These results are derived from this fungal species and a hypothesis that involves further research on other plant pathogens to demonstrate the overall potency of essential oils. This study references the easy, economic, and environmental management and control of plant diseases using essential oils and byproducts.

## Introduction

Ginger (*Zingiber officinale* Roscoe) is identified as an ancient spice plant often used by traditional Chinese medicines, Ayurvedic, eastern, Tibb-Unani, and herbal drugs. It belongs to the family Zingiberaceae and is generally consumed as fresh and/or dried seasoning for cooking^1^. Ginger is commonly used as a home remedy for the treatment of digestive disorders, cough, cold cardiac problems, and various health issues. In addition, ginger is the best antioxidant and possesses potential against inflammation and muscular pain; it also shows wound healing, antiseptic, antimicrobial, and mosquito repellent activities^2^. Recently, essential oils extracted from ginger have been extensively used against many human and plant pathogens^3^. The most common bioactive compounds in ginger essential oil are citral, zingiberene, thujene, pinene, camphene, sabinene, pinene, myrcene, and limonene, which possess versatile properties and advantages^4^.

Essential oils (EOs), sometimes called essence, flavor, or volatile oils, are basically secondary metabolites secreted by organisms, particularly plants^5^. Most of the EOs are extracted from plant materials using distillation, pressurization, extraction, or appropriate solvents^6^. EOs are frequently used for perfumes, cosmetics, food items, and medicinal purposes^7^. The chemical nature of these EOs shows inconsistency subjected to the planting area and environmental conditions where they are produced or extracted^8^. Furthermore, the composition of EO could be affected by the materials and methods employed for drying, extraction, and hydrodistillation^9,10^. In addition to their pleasant aroma, EOs are also well known for their biologically active components and features in various applications^11^. Historically, essential oils were extracted by classic distillation equipment^12^, which may have impaired the oil quality and quantity as chances for losing certain volatile components during the process, poor extraction efficiency, a lack of solvents for extracting unsaturated compounds, and, most importantly, temperature variations and other environmental conditions^13^.

Microwave assisted hydrodistillation is a unique and advanced extraction technique, recently gained too much attraction and is ideal for compact size, quick operation, swift control, molecular heating processes, and solvent-free extraction^14^. Is highly efficient, safe, economical, low-cost, time saving, chemical and solvent free and is especially termed green technology^15^.

Taro *Colocasia esculenta* (L.) is one of the ancient cultivated tuber crops belonging to the Araceae family^16^. It is cultivated worldwide, especially in Africa, China, America, the Indian subcontinent and the West Indies^17^. It is a common staple food across Southeast Asia, China, and the United States, particularly Africa^18^. Taro contains a high content of carbohydrates, minerals, protein, and vitamins^19^. It possesses medicinal potential against pathogenic and bacterial infections^20^. Despite this, taro itself is susceptible to various plant diseases and pests; among them, leaf blight of taro caused by the oomycete *Phytophthora colocasiae* is most destructive, causing huge yield losses around the world. The disease appears mostly on aerial parts of the plant and is responsible for destroying taro leaves in a short period. The pathogen has a complex life cycle based on growth characteristics as semi-biotrophic parasitic and necrotrophic stages^21^. The optimum temperature for disease infection and epidemics is 25–30 °C, and a relative humidity above 90% is conducive to the germination of zoospores. The pathogen is a heterogeneous species that asexually produces sporangia, sporangiospores, and zoospores. In contrast, as a result of sexual reproduction, it produces oospores (thick-walled sexual spores) that survive for a long period of time in plant residues and corms retained for next season propagation^22^. The cell walls of zoospores are usually expanded to develop thick-walled spores (chlamydospores) that undergo dormant conditions to promote survivability in soil and corms^23^. Due to this phenomenon, Taro disease has become the key pathogen, and continuous cultivation promotes pathogenic biomass accumulation within the soil.

Pathogen germinates rapidly under favorable conditions and quickly spread to adjacent plants, especially in the rainy season, leading to a sharp decline or even death of crops, resulting in 100% loss^24^. Farmers often use extremely toxic systemic fungicides such as (Metalaxyl, Hymexazol, benomyl, cyproconazole, azoxystrobin difenoconazole, carbendazim, and propiconazole) to combat TLB diseases, which is efficient in acute, but long-lasting consequences of these chemicals is still a great concern. Recently, there has been a tendency to adopt chemical-free items, particularly nutrition products from organic agriculture. As a result, scientists pay more attention to biological fungicides and phytochemicals because they are ecologically safe, long-lasting, and effective alternatives for controlling various destructive plant diseases.

Ginger is inexpensive average price (USD 1/Kg) and easily available with high oil yield and extensive biological properties. We conducted this study using ginger essential oil as an impending alternative to toxic antimycotics. Ginger essential oil is well known for its antimicrobial properties and is applied as a natural solution to manage and avert various plant pathogens^25^. Even though, EO of ginger is recommended for many other pathogens, it’s the first attempt to combat TLB pathogen. This study was designed to describe a safe and easy-to-use technique for the extraction of essential oils that may be used for—eco-friendly control of plant diseases, thus meeting clean, green, and sustainable agricultural goals.

## Results and discussion

### Chemical analysis of ginger essential oil

**Figure. 1:**
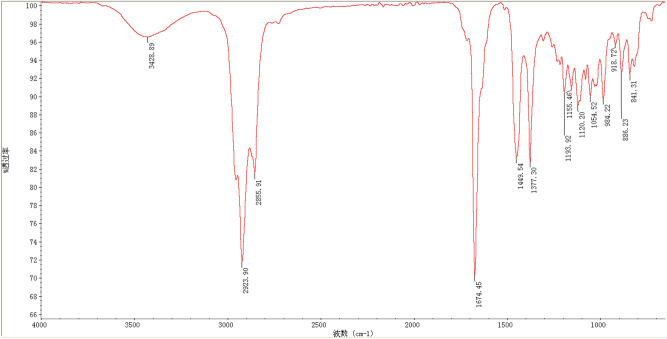
FTIR spectra of ginger essential oil.

The essential oil was extracted from ginger rhizomes by the MAHD method with a yield of 2.5%. EO was analyzed, and we found Citral_z+e_; 3,7-dimethyl 1-2,6 octadienal C_10_H_16_O_1_ (95%) as the major oil compound (Fig. 1). Ginger essential oil compositions from various previous studies are also available^26^. Ginger has been widely studied for essential oil and component applications in various food preservatives, disinfectants, therapeutics, and nontoxic antimicrobial products^27^. Ginger EO has different levels of hydrocarbons, and the main components reported are citral and zingiberene extracted from the rhizome^28^; however, variations in the chemistry and yield of EO can be due to different biotic and abiotic factors. Ginger essential oil is widely used as an antifungal and antibacterial agent in numerous ways, such as synergistic effects when supplemented with other biological compounds. Therefore, ginger oil has many characteristics, which determine its importance in different industries^29^.

### Effect of ginger EO on radial colony growth inhibition

The results showed that increasing the concentration of ginger essential oil significantly inhibited the radial growth of the fungal mycelium (P ≤ 0.05) (Table 1). The minimal inhibitory concentration (MIC) for the growth of the radial fungal colony was 1250 ppm and beyond. At the same time, metalaxyl inhibited 100% growth of the fungal colony at a 0.15 mg/mL concentration under the similar conditions. Pre-inoculated mycelium plugs from treatments causing complete mycelial inhibition were removed and transferred onto new PDA plates to confirm the viability of the mycelium, which was not able to grow again and was considered as dead.

**Table 1:** Mycelium, zoospores, and sporangium inhibition of *P. colocasiae* by ginger EO.

EO concentration (ppm)	Radius colony (mm)	Inhibition %*		
		Mycelium	Zoospore	Sporangium
0.00	45.00 ± 0.0a	0.00 ± 0.00e	0.00 ± 0.00d	0.00 ± 0.00d
156	28.00 ± 1.63b	37.77 ± 1.81d	59.42 ± 0.91c	77.06 ± 0.68c
312	10.75 ± 1.73c	76.11 ± 1.89c	82.49 ± 0.68b	83.20 ± 0.51b
625	5.50 ± 1.08d	87.77 ± 1.20b	100.00 ± 0.00a	100.00 ± 0.00a
1250	0.00 ± 0.00e	100.00 ± 0.00a	100.00 ± 0.00a	100.00 ± 0.00a
2500	0.00 ± 0.00e	100.00 ± 0.00a	100.00 ± 0.00a	100.00 ± 0.00a
5000	0.00 ± 0.00e	100.00 ± 0.00a	100.00 ± 0.00a	100.00 ± 0.00a
Metalaxyl	0.00 ± 0.00e	100.00 ± 0.00a	100.00 ± 0.00a	100.00 ± 0.00a

Values indicate means of four replications, and means followed by different subscripts in the same column indicate significantly different based on the LSD (P < 0.05).

*Inhibition percentage compared with negative and positive control.

Although this was the first trial to investigate the fungicidal properties of ginger EO against taro leaf blight, previous literature has described various antimicrobial and fungicidal properties of this oil. Ginger oil has multifaceted chemical constituents that involve both main and minor compounds relevant to the antifungal mode of action. In this study, citral was evaluated as the most important constituent of ginger EO, which has been identified as a precarious compound^30^. Various studies defined it as inhibiting several bacterial and fungal pathogens by reducing interactions of ATPase, alteration of mitochondrial structure, deterioration of cell wall and membranes, and cell structure and metabolism^31^. In addition, several studies have shown that citral has antifungal and antibacterial potential against food-contaminating microbes^32^ and can be used as a natural, long-lasting food preservative. Nevertheless, the effect of trace components in ginger oil is not negligible and may not be disregarded. In this respect^33^, suggested that the antifungal activity of EOs might also be associated with synergetic effects of primary and secondary metabolic compounds.

At the same time, in this sense, the volatile compounds in ginger may inhibit the growth of the fungi. The inclusive efficacy of citral is also described for bread preservation against *Aspergillus niger*^34^, application against many foodborne pathogens^30^, and fish rotting pathogens^35^.

### Ginger EO against sporangia and zoospore germination

As per our results, with a consistent increase in EO concentration, a subsequent decrease in the germination of zoospores and sporangia was observed (Table 1). The minimum inhibitory concentration for sporangia and zoospores was 625 ppm. However, the morphology of the sporangia and zoospores was slightly deformed, as observed under the microscope. This is proof of spore cell wall lysis induced by volatile components of ginger oil. Previous studies have revealed the antifungal effects of eucalyptus essential oil in combating the germination of *P. colocasiae* zoospores. Our results are comparable to those of Sameza (2014), as zoospores lack biological walls, whereas they exist in sporangia^36^. Under favorable conditions, zoospores germinate, lose Flagellum, and encyst in cell wall-like structures, creating germ tubes that trigger the infection process^37^. Lipophilic molecules of essential oils may restrict this encapsulation and cell wall synthesis that hinders the germination of sporangia^38^.

Alternatively, the trace components of essential oils inhibit germination through a synergistic effect because their hydrophobic properties allow them to penetrate cell membranes. EO can disturb some enzyme functions involving spore germination by prolonging the lag phase in the course of spore germination and cytoplasmic disruption in fungal hyphae, causing thinner, distorted and damaged hyphal walls; the cell wall was observed in *Cladosporium cladosporioides*, *Trichoderma viride*, and two other molds from *Alternaria*^39^*.* Disease establishment and dissemination of mycological pathogens mostly occur through spore propagation; consequently, inhibition of spore germination is an anticipated approach of targeting a pathogen to inhibit or slow down inter- and intra-plant disease spread^40^.

### Reduction of leaf necrosis by ginger essential oil

After a 3-day incubation time, some visible symptoms of necrosis were observed on leaves in the negative control treatments, and certain concentrations of ginger oil were applied at 156, 312, and 625 ppm (Table 2). In contrast, inoculated leaves sprayed with 156 ppm metalaxyl and essential oil concentrations (1250 ppm) or higher did not show any noticeable symptoms. The results shown that an increase in essential oils concentration significantly decrease the leaf necrosis symptoms (Table 2). As mentioned earlier, zoospores are the basic life cycle stage of *P. colocasiae* and responsible for initiating primary infection. Our results could be an outcome of fungicidal components present in ginger essential oil, which lyse zoospores or hinder their germination. Filomena Nazaro (2017) described the ability of essential oils to enhance plant disease resistance. The resistance mechanism is thought to respond to the acute release of H_2_O_2,_ resulting in oxidative stress. This is exactly what happens when plants are under microbial or abiotic stress^41^. As mentioned earlier, citral is an integral part of ginger essential oil, and it has been reported to reduce the virulence of many pathogens (inhibition of the establishment and adhesion of reproductive structures, phospholipid hydrolysis, and protease activity^29^. This effect may be related to the role of these molecules in the ATPase-dependent emanation process^42^. It also prevents mycelial development and the production of aflatoxin, which leads to irreparable changes in the mycelium structure, reduction in cytoplasm and mitochondria dysfunction^43^. These compounds also interfere in electron transmission and interact with ionic channel proteins on the surface of mycelial membrane which may consequently modify the membranes permeability leading to cytoplasm leakage. This phenomenon triggers the molecules linked to the REDOX signaling mechanism that regulates many cell functions^44^. The results show that curative treatment is more effective in preventing leaf necrosis with EO application.

**Table 2:** Inhibition effects of ginger EO on leaf blight and sporulation of, *P colocasiae.*

Essential oil	Latency time	Leaf blight symptoms		Sporangia production	
Concentrations (ppm)	Hour	Diameter (mm)	Inhibition* %	10^3^ Sporangia/mL	Inhibition* %
0.00	**72**	16.50 ± 0.79a	0.00 ± 0.00e	95.00 ± 1.29a	0.00 ± 0.00e
156	**72**	8.92 ± 0.52b	45.90 ± 3.19d	38.25 ± 1.49b	59.73 ± 1.57d
312	**72**	5.85 ± 0.28c	64.54 ± 1.72c	24.50 ± 1.19c	74.21 ± 1.25c
625	**72**	3.00 ± 0.27d	81.81 ± 1.69b	12.75 ± 0.85d	86.57 ± 0.89b
1250	=	0.00 ± 0.00e	100 ± 0.00a	0.00 ± 0.00e	100 ± 0.00a
2500	=	0.00 ± 0.00e	100 ± 0.00a	0.00 ± 0.00e	100 ± 0.00a
5000	=	0.00 ± 0.00e	100 ± 0.00a	0.00 ± 0.00e	100 ± 0.00a
Metalaxyl	=	0.00 ± 0.00e	100 ± 0.00a	0.00 ± 0.00e	100 ± 0.00a

Significant values are in bold.

Values indicate means of four replications, and dissimilar letters in the same column indicate significant differences based on the LSD test (P < 0.05).

*Inhibition percentage compared with the control without ginger EO = Indicates no symptoms.

### Ginger EO against sporangia production in leaf assay

As the concentration of essential oil increased, the formation of sporangia decreased significantly (P ≤ 0.05) (Table 2). The maximum inhibition of sporangia was recorded at 1250 ppm with ginger essential oil and metalaxyl treatment as 100% inhibition. Furthermore, when the oil concentration was elevated, some deformities in the morphology of the sporangia witnessed. Ginger EOs contain phytochemicals that have antifungal properties against a wide range of phytopathogenic fungi. These compounds have been shown to inhibit disease invasion and degrade cell walls and biofilms due to their high permeability and percolation while interfering with cell membrane function^45^. The lipidic components of EO enables them to enter the biological membranes, interfering with enzymes that ultimately malfunction and produce uncontrolled cell wall synthesis^46^.

Moreover, EO contains several volatile chemicals that may induce cell lysis, impairing sporulation. Several investigations have shown that ginger essential oil has an inhibiting impact on fungal infections^47^. At the same time, several volatile chemicals in essential oils are considered to inhibit sporangia development. *Eucalyptus globulus* EO at 625 ppm^36^ and *Citrus aurantifolia* EO at 800 ppm inhibited the sporangia production in *Phytophthora colocasiae*^48^.

### Reduction of symptoms on taro corms inoculated with fungus

**Figure 2:**
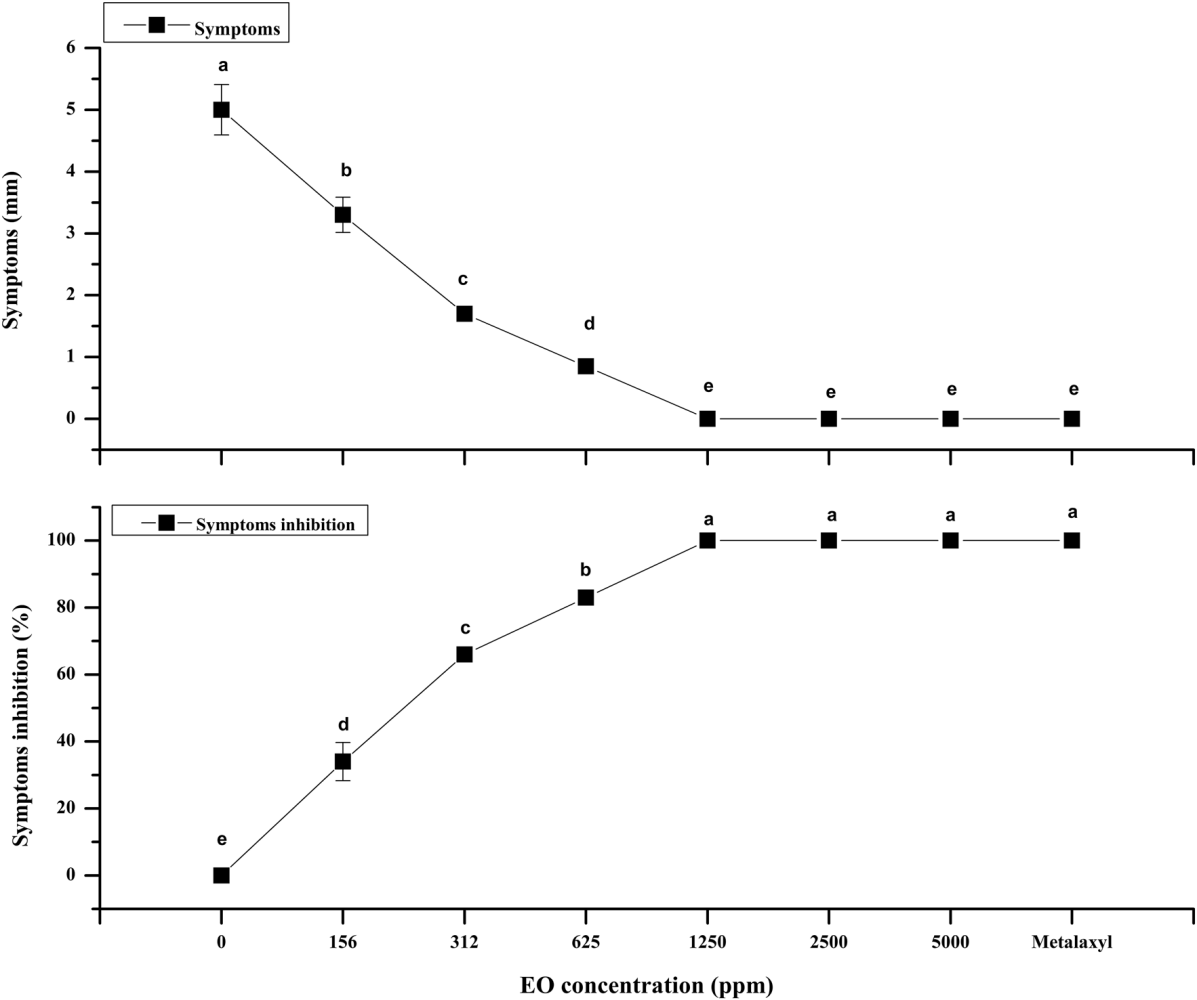
Inhibition effects of ginger EO on taro corms inoculated with *P. colocasiae***.** Values are the means of four replicates, and dissimilar letters indicate significantly different based on the LSD test.

The mycelial discs inserted into taro corms began infestation 7 days after inoculation, whereas negative control treatments and certain EO treated corms i.e., 1250 ppm or lower concentrations presented characteristic deterioration symptoms. Dark brown spots appeared around the inoculation sites and the surface of corm was apparently impaired. In contrary Metalaxyl treatment and higher concentration of essential oil treatments i.e., 2500 ppm and above did not show any visible symptoms on inoculated corms (Fig. 2). Inhibition of infection in taro corms probably because of volatility of EOs that enabled quick absorbance in fibrous tissues of corms. In addition to blight symptoms on leaf, (TLB), disease is also a major cause of severe deterioration in corms after harvest and storage^49^. In contrast, oospores hibernate on the surface of corms for a long time and act as the main inoculant, causing primary infection and leading to epidemics. Considering the fact that recently EOs are commonly used to disinfect perishable fruits and vegetables during storage for prolonged shelf life^50^, various techniques for the prevention and curation characteristics of plant essential oils have been studied for the economic, efficient and effective treatment of postharvest diseases^51^.

## Conclusion

In the context of returning to nature, with the innovation of science and technology for improved living standards, people have begun to seek solutions for food hygiene without chemical additives. Therefore, biological pesticides and plant-oriented chemicals have received special attention from researchers because they are environmentally friendly and nonhazardous, sustainable, and effective alternatives against many noxious phytopathogens. Considering the perilous nature and direct/indirect paraphernalia of systemic fungicides on human and environmental health. At the same time, ginger is inexpensive and easily available with high oil yield and possesses extensive biological properties. We conducted this preliminary study using ginger essential oil as an impending alternative for toxic fungicides. Our results are derived from this fungal species and a hypothesis that involves further research on other plant pathogens to demonstrate the overall potency of essential oils. This study references the easy, economic, sustainable, and eco-friendly management and control of plant diseases using essential oils and byproducts.

## Materials and methods

### Extraction of essential oil

**Figure 3:**
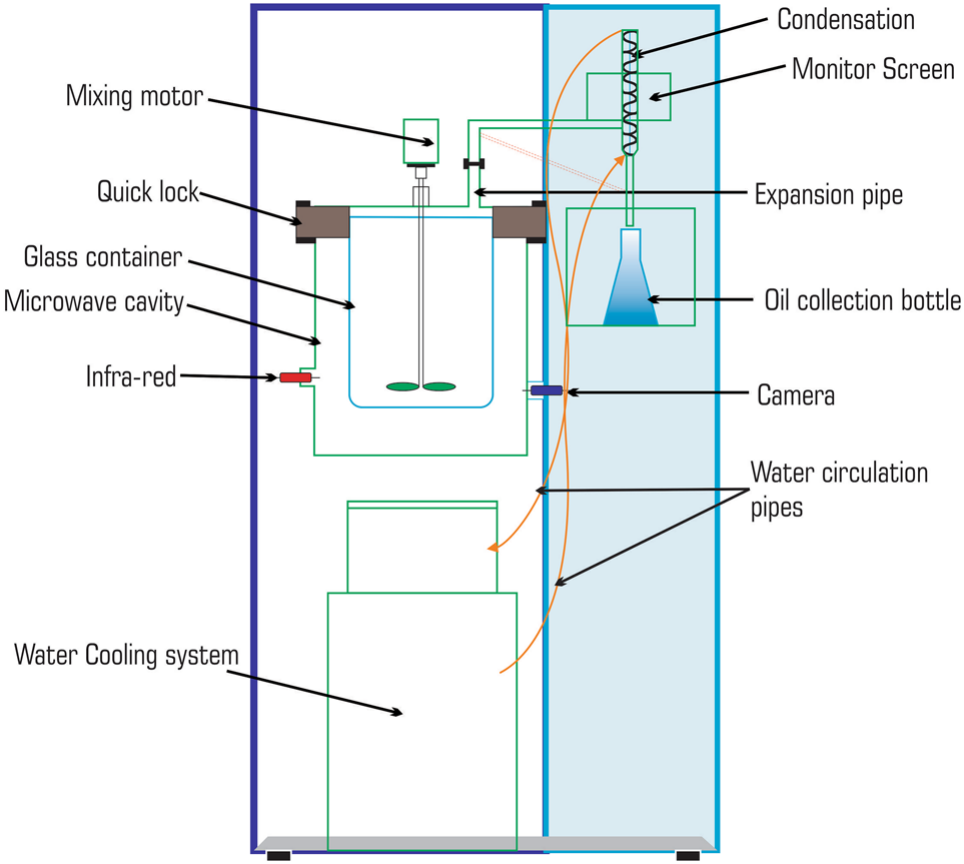
Microwave-assisted hydrodistillation equipment design.

Fresh ginger rhizomes were harvested with permission from horticulture department, Southwest University of Science and Technology in Mianyang, Sichuan Province of China, during January 2021. Rhizomes were dried at room temperature and cut into small pieces with a kitchen knife before extraction. Essential oils were extracted with a modified microwave-assisted hydrodistillation machine designed with 800-Watt microwave power, highest power input, (1000 Watt) resonant frequency (2.400 GHz) internal microwave generator voltage (420 VAC) and a working temperature range of 100–300 °C. Equipped with a 5-L glass container for heating plant material mounted with a Clevenger system connected with leak-proof water circulation pipes. The instrument provides a self-water cooling technology and a condensation loop to avoid excessive use of tap water. A device for mechanical mixing is also installed that homogenizes the plant material during extraction. PLC with a color touch screen, convenient for use, and a closed-loop PID automatically controls microwave power, temperature, and working duration, with programming for different plant extractions (Fig. 3). For extraction, 200 g of ginger pieces were presoaked for 1 h in 300 mL distilled water and boiled at 100 °C for 30 min and then 150 °C for the next 30 min, and the process was repeated three times to obtain the maximum oil yield. The oil floating on surface of the aqueous distillate was parted from the latter and dried over anhydrous sodium sulfate. EO was kept in 2.5 ml opaque glass bottles enfolded with aluminum foil and stored at 4 °C in the refrigerator for further application.

### Essential oil chemical analysis

A PerkinElmer USA Spectrum Two™ FT-IR spectrometer with analytical equipment was used to perform FTIR spectroscopy with the ATR (attenuated total reflectance) technique to identify the major components of EO. Aldrich FTIR Collection Second Edition software was used to analyze the spectra (Thermo Fisher Scientific Inc.). A small drop of EO sample was placed on ATR crystal of FTIR spectrometer using a glass dropper, and the spectra and Peaks were identified and observed on a computer screen. The data were taken three times for authenticated results.

### Pathogen isolation

**Figure 4:**
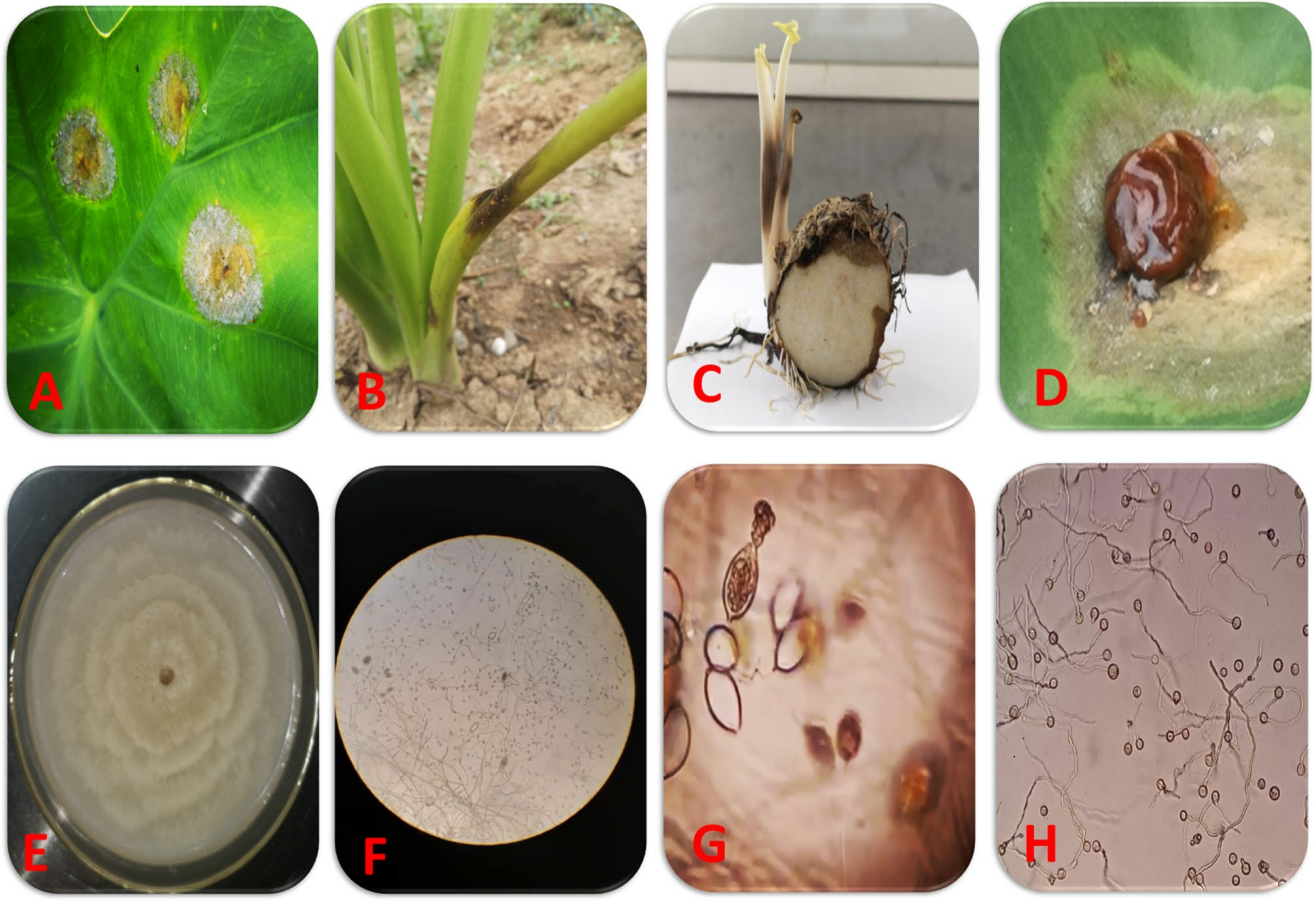
Typical Symptoms of taro leaf blight and fungal Morphology of *Phytophthora colocasiae*. (**A**) Leaf blight lesions showing silvery rings of sporangia, (**B**) Symptoms on leaf petioles, (**C**) Cross-section of corm showing decaying symptoms, (**D**) Typical symptoms, lesion showing plant sap exudates, (**E**) Colony morphology of *P colocasiae*, (**F**) Sporangia with mycelium and zoospores, (**G**) sporangia releasing zoospores, (**H**) Zoospores magnified under compound microscope.

The pathogen was cultured from lesions of infected leaves with typical blight symptoms, i.e., silvery rings of sporangia and plant sap exudates near necrotic leaf areas, petioles, and corms (Fig. 4A–D). The leaves showing disease symptoms were sliced into 2–5 mm^2^ pieces from margins of infected parts adjacent to healthy tissues. The leaf pieces were surface sterilized with 0.1% mercuric chloride for 1 min and then washed twice with sterile distilled water. After drying on sterilized blotting paper, the leaf pieces were then transferred to petridish containing fresh potato dextrose agar (PDA) medium and kept for incubation at 25 °C. Immediately after mycelia appeared on culture plates, it was inoculated to new PDA culture plates to maintain pure culture of fungus (Fig. 4E). The isolated fungus was identified as *Phytophthora colocasiae* based on colony morphology, sporangia shape, zoospores, and microscopic observation of mycelium (Fig. 4F–H), Pathogenicity established by recapitulation of isolated pathogens to satisfy Koch's postulates. Pathogen isolates were maintained at 5 °C in a refrigerator and sub-cultured periodically on fresh PDA medium. Sporangium production was induced by inoculation of five culture slants (5 mm) with 10 ml sterilized distilled water in test tubes for 3 days under a bright fluorescent lamp. For the release of zoospores, sporangia were chilled at 4 °C for half an hour and then shifted to 25 °C for 15 min as described by Nath^52^. Zoospores separated from sporangia by sieving with thin filter paper. The volumes of Sporangia and zoospores were calibrated to 10^3^/ml.

### Fungicidal assessment of ginger EO

#### Mycelium growth inhibition (MGI) assay

Mycelial growth inhibition was evaluated using the poisoned food technique as described by Ali^53^ with little modification. The oil was diluted at a Tween 80 to 90:10 (V/V) ratio as a stock solution; a final concentration of 5000, 2500, 1250, 625, 312, and 156 ppm was supplemented with PDA at 50 °C before pouring in Petri dishes. Ten milliliters of PDA medium was poured into every 9 cm sterilized petridish, and each treatment was repeated four times. PDA medium containing only Tween 80 served as a negative control, and a minimum concentration of 156 ppm metalaxyl was added to PDA as a positive control. In the control treatment, only PDA was retained, which was used to compare the growth in all treatments. A 5 mm disc from the edge of actively growing mycelium of test fungus was inoculated and kept for incubation at 25 °C. When the controlled Petri dishes were fully grown, the antifungal potential of the essential oils was evaluated^36^. Mycelial growth inhibition (MGI) was determined by reducing the radial colony growth diameter of the treatment plate (X_t_) from the negative control petri dish (X_ck_). (MGI = X_ck_ − X_t_). From complete or partial mycelial inhibition treatments, mycelium discs already inoculated on treatment plates were transferred to fresh unamended PDA plates to confirm viability of inoculated mycelium after treatment with EO.

### Effect of ginger EO on sporangia production

The production of sporangia was measured by liquid dilution method using eight vegetable juice broth (V8) medium amended with EO concentrations as described before for mycelium inhibition assays (adapted from Tchameni 2018)^48^. Ten discs of 5 mm from 7 days actively growing culture of the test fungus were mixed in (10 ml) sterilized distilled water and centrifugated at 4000 rpm (5 min) for homogenization. A hemocytometer was used to count spores from 10 μL of precipitation. The formula *I*_*s*_ = *(N*_*0*_ − *N*_*t*_*)/N*_*t*_ × 100 was used to evaluate spore inhibition (*I*_*s*_), where *N*_*t*_ is the oil treatment and compared with the control *N*_*0*_ treatment. Zoospore release was counted after 3 h, and sporangia formation was observed after 24 h as described by (Sameza^36^). The metalaxyl treatment was retained as an active control and treated with Tween 80, and media without treatment served as the negative control.

### Inhibition of sporangia and zoospore germination

A slightly modified technique (adopted from Sameza 2014)^36^ was used to estimate zoospore germination. The essential oils as per concentrations described earlier were dissolved in tween 80 whereas metalaxyl was dissolved in sterile distilled water, and homogenized with (V8) broth, and transferred to test tubes. One-week-old pure culture was inoculated into tubes with 500 μL pathogen suspension pre-adjusted to 10^6^ cells/mL and incubated at 25 °C. Each treatment was repeated three times, and this process was repeated twice. Three hours later, the percentage of zoospore germination was counted with a hemocytometer under a microscope, and sporangia were observed the day after inoculation.

### Assessment of necrosis and sporangia production on leaves

Mature taro leaves detached from healthy plants (TLB Susceptible variety Chuankui 1) were inoculated under controlled sterile conditions in an incubator. Before inoculation, leaves were sprayed with essential oil concentrations as described earlier. The metalaxyl used as an active control and treated with only sterile distilled water served as the negative control. After 1 h of treatment, 5 mm discs cut from active growing mycelium culture of fungus was inoculated on the adaxial surface of leaves. Four leaves were inoculated for each treatment, and the experiment was repeated twice. Inoculated leaves were immediately covered in black polythene bags to ensure moisture, and incubated in 90% humidity for 3 days and monitored every day until characteristic symptoms appeared in negative control. Disease evaluation was based on observation of latency period (time until symptoms appeared), diameter of necrotic area and sporangia count on the leaves. Reduction of disease incidence (% RDI) of EOs was evaluated using following formula: *RDI* = *(D*_0_ − *D*_*x*_*/D*_*0*_*)* × 100, where *D*_*x*_ is the lesion diameter with essential oil treatment, and *D*_*0*_ is the negative control.

### Antifungal assay of EO on taro corms

Selected taro corms of uniform shape, size, (approximately 200 gm each) bearing no visible signs of damage or substantial distortion were used for the trial. The corms were rinsed under tap water, and immersed in 1% sodium hypochlorite (NaOCl) for 1 min and then washed twice with sterile distilled water for 5 min for surface sterilization. The corms were then submerged to EO for 1 h at different concentrations as previously described, and metalaxyl was used as a control. The previously isolated fungus *P. colocasiae* was used as inoculum. Taro corms were inoculated by removing three 5 mm deep plugs from the upper, middle, and lower sites of corms. The boreholes were inoculated with 5 mm PDA plugs obtained from margin of active growing culture of *P. colocasiae*. The boreholes were sealed with the same plugs removed from same site of corm. Each treatment consisted four replications whereas Metalaxyl, served as positive control and no EO as negative control, and the trial repeated twice. Taro corms were kept in a dark humid incubator at 25 °C for one week; then, the corms were cut vertically at inoculation site to check fungus infection.

### Analysis of data

Data was analyzed for homogeneity of variance using IBM (SPSS Statistics software package 23 version). Results were described subjected to means (±) standard deviation (SD), analysis of variance (ANOVA) to identify the correlation across treatments performed using Tukey's HSD test. The least significant difference (LSD) was used to assume significantly different means at significance level of (P < 0.05). MS excel 2019 used for data computations for percentages and plot the graphs.

### Ethical approval

This study complies with relevant institutional, national, and international guidelines and legislation.

### Permissions

Appropriate Permissions have been obtained for plant specimens used in present study.

## Data Availability

The datasets used and/or analyzed during the current study are available from the corresponding author upon reasonable request.
